# Authors’ response to Graham Rook’s commentary

**DOI:** 10.1093/emph/eoab012

**Published:** 2021-04-11

**Authors:** William Parker, Joshua T Sarafian, Sherryl A Broverman, Jon D Laman

**Affiliations:** 1 Department of Surgery, Duke University Medical Center, Durham, NC, USA; 2 Department of Biology and the Duke Global Health Institute, Duke University, Durham, NC, USA; 3 Department of BSCS, University Medical Center Groningen, Groningen, The Netherlands

We thank Professor Emeritus Graham Rook for his Commentary on our recent article in *EMPH* and thank the editors for this opportunity to respond. He sees problems with several aspects of our work, and we will address the key points as space allows.

A major concern raised by Rook is our assertion that complex eukaryotic symbionts, particularly helminths and protists, constitute a keystone cohort of species central to immune function in humans. Rook was one of the pioneers of this idea, and, as he points out, has now changed his opinion. He asserts that different helminths regulate immune function in different ways, so therefore, ‘there is no constant inevitable helminth-associated factor that could drive hard-wired genetic dependence on helminths’. Importantly, Rook is not refuting the prevailing view that symbiotic helminths and protists have been ubiquitous during the past 350 million years of evolution, and he agrees that symbiotic codependence evolves from inevitable symbiotic associations. However, he does argue that, because exposure to particular helminths and protists is not consistent, no absolute codependence is likely to emerge.

This argument is untenable. Studies from Argentina by Correale and colleagues, acknowledged and accepted by Rook, show that three groups of complex eukaryotic symbionts, roundworms, flatworms and protists, all effectively block the progression of multiple sclerosis (MS). Correale’s work provides strong support for the view that these three phylogenetically distinct groups of organisms have evolved convergently in a manner that allows them (i) to survive in a vertebrate intestine and (ii) to modulate host immune function in similar ways. This view should not be surprising given that examples of convergent evolution abound, including the well-known achievement of flight by insects, birds and bats.

Further, as predicted by Flowers and Hopkins [1], the systematic study of the failures and successes of more than a thousand individuals self-treating with various species of helminths has provided considerable insight into the effects of helminths on human immune function and health. Contrary to the assertion by Rook, results from these systematic studies are not anecdotal in nature, except on occasion where appropriate for the study design. These studies conclusively affirm results from Correale’s studies, providing clear explanations for differences between the impressive results of natural exposures reported by Correale and the negative results of several clinical trials [2]. These issues were reviewed in our original manuscript, and hence are not recounted here.

Rook is also concerned about our prediction that less severe reactions to SARS-CoV-2 infection can be expected in parts of the world with endemic helminth exposure. This prediction is based on the observations that (i) severe reactions to SARS-CoV-2 are associated with autoimmune-like reactions in areas where the human population is essentially devoid of helminths and protists, and (ii) autoimmune diseases are much less prevalent in areas with endemic helminth exposure. A caveat to this prediction is that lethal reactions to SARS-CoV-2 infection might be quantitively high but qualitatively different in helminth endemic communities. Importantly, this caveat does not diminish the possibility that, just as helminths reduce the incidence of autoimmune disease, they may also prevent autoimmune-like reactions to SARS-CoV-2 that result in mortality or severe morbidity.

We agree with Rook that nothing is absolutely proven with regards to the severity of SARS-CoV-2 infections in parts of the world with endemic helminth exposure. Nevertheless, at the present time, reports from the field are consistent with our prediction and indicate that suboptimal systems hygiene may be an important factor in reducing the age-adjusted mortality per infection with SARS-CoV-2. More importantly, we are confident in our assertion that the absence of a keystone cohort of species comprised of helminths and protists is a significant contributor to chronic, pathologic immune reactions in affected cultures.

Rook asserts that alterations in the microbiota should be considered as a cause of autoimmune disease. He points out that the tuberculosis vaccine, the BCG vaccine, has been associated with better outcomes in SARS-CoV-2 infections, and that increased amounts of latent tuberculosis rather than increased amounts of helminths may, as an alternative hypothesis, account for less autoimmune disease. In response, we applaud the observation that the BCG vaccine has unexpected benefits related to COVID-19. Indeed, one of us has reported unexpected beneficial effects of vaccines in another setting [3]. That being said, latent tuberculosis infections, *per se*, are associated with more rather than less autoimmune disease, and the infectious disease caused by the tuberculosis organism is caused by denser living conditions, an evolutionary mismatch resulting from the agricultural revolution. Thus, more rather than less tuberculosis is considered to be caused by evolutionary mismatch. Considerations of tuberculosis aside, we agree with Rook that alterations of the microbiota should be considered as a potential cause of autoimmunity. However, conclusive data regarding this issue are lacking. For example, we strongly agree (manuscript in preparation) with the view of Swidsinksi *et al*. [4], who argue convincingly that, despite extensive study, current data do not support a causal link between the microbiota and MS.

The widespread implementation of systems hygiene is necessary to avoid pandemics of infectious disease. As described in our original paper, this type of hygiene results in an almost complete loss of protists and helminths from the ecosystem of the human body, a condition that unfortunately contributes to allergy, autoimmune disease, digestive disorders, and probably several neuropsychiatric conditions. Rook argues that the regulatory hurdles to re-introducing benign (non-pathogenic) helminths are ‘overwhelming’, but difficulty should not cause us to abandon work aimed at the treatment and prevention of disease. More importantly, one of us (WP) who is actively pursuing regulatory approval for therapeutic use of helminths has observed that regulatory issues are not the problem as much as a lack of financial incentive for forward progress [5]. We would argue that, if anything is overwhelming, it is the burden of disease caused by aberrant immune function in areas without complex eukaryotic symbionts. With that in mind, it is important for stakeholders to take action and test the possibility that the reintroduction of benign helminths may alleviate a wide range of immune-associated pathology, perhaps including many severe adverse reactions to the SARS-CoV-2 virus.

**Conflict of interest:** None of the authors have a financial conflict of interest. Duke University Medical Center has a financial interest in technology involving helminth therapy.
